# New Genera and Species Records of Nicaraguan Eumolpinae (Coleoptera: Chrysomelidae) Including a New Species in a New Generic Record for Central America

**DOI:** 10.1007/s13744-022-00987-2

**Published:** 2022-08-19

**Authors:** Jesús Gómez-Zurita, Jean-Michel Maes

**Affiliations:** 1 Institut Botànic de Barcelona (CSIC-Ajuntament de Barcelona), Barcelona, Spain; 2 Museo Entomológico de León, León, Nicaragua

**Keywords:** Biodiversity, New species, New record, Taxonomy, Tropical dry forest

## Abstract

In this work, we have examined a large sample of Eumolpinae leaf beetles from Nicaragua and found 18 species reported for the first time in this country, including the new species *Caryonoda funebris* **n. sp.**, which also represents a new genus record for Central America, and two genera of Typophorini not reported from Nicaragua so far: *Paria* LeConte, 1858 and *Tijucana* Bechyné, 1957. Apart from the description of the new species and taxonomic commentaries on each of the new country records, we also illustrate these species along with drawings of male genitalia and spermathecae when available to assist the interpretation of our taxonomic decisions in the future. We take the opportunity in this work to formalize the combination of *Chrysodina cupriceps* Lefèvre, 1877 as *Chrysodinopsis cupriceps* (Lefèvre) **n. comb.**

## Introduction

Our knowledge of Central American Eumolpinae, or Chrysomelidae in general, is still much dependent on the monumental work by Martin Jacoby for the encyclopedia *Biologia Centrali-Americana* (Jacoby 1880-1892), who also updated and commented on species described by previous authors, mainly Édouard Lefèvre, who had studied several species from Central America (e.g., Lefèvre 1875, 1877, 1885). Much later, Jan Bechyné made some significant contributions, not only describing new species, but also refining the systematics of the group, mostly in the 50s of the last century (e.g., Bechyné 1951, 1953, 1954), but this entomologist was mainly focused on the South American fauna, even though he occasionally discussed Central American species in his massive studies. We owe him the most comprehensive checklist of Neotropical, or more accurately Central and South American Eumolpinae (Bechyné 1953), which has been the basis for subsequent catalogs in the region, including the refined compilation by Flowers (1996). Through the 50s and up to the 70s of the past century, Doris H. Blake expanded the study of Nearctic species of Eumolpinae to their Central American and Caribbean relatives, and we owe her some in-depth generic revisions with the generalized use of male genitalia to assist species recognition for the first time in this group (e.g., Blake 1970, 1976a, b). Finally, modern systematics of Central American Eumolpinae has been in the hands of R. W. Flowers, motivated by the need to understand and describe the diversity of Costa Rican Eumolpinae (e.g., Flowers 1997, 2003, 2004, 2018), which pressed him to take a regional look on the matter as well (Flowers 1996). In all these studies, Nicaragua has occupied a marginal position, with comparatively limited collection material available for study, compared with Costa Rica or Mexico, for example, which in great part came from the expeditions of T. Belt, E. M. Janson, and W. B. Richardson for *Biologia Centrali-Americana* in the late nineteenth century (Godman and Salvin 1918). This material allowed for the description of several new species and refined our knowledge about the distribution of others, but in general, our understanding about the Nicaraguan fauna of Eumolpinae is still preliminary and very fragmented (Maes and Staines 1991).

In the course of preparation and study of a large sample of Eumolpinae from Nicaragua, we came across a number of new species and even generic records for the country. Among them, it stood out one specimen clearly belonging to the genus *Caryonoda* Bechyné 1951, which was only known from South America (Bechyné 1953; Bechyné and Springlová de Bechyné 1961). In his review of Central American Eumolpinae, Flowers (1996) mentioned observations of *Caryonoda* from Panama and Costa Rica perhaps consistent with more than one species, but they were not described nor given other details about their identities. *Caryonoda* is a small genus of Neotropical Eumolpinae that was proposed based on a suite of very unique characters, most notably the shape of prosternum, with sides of prosternal process flanked by longitudinal grooves anterior to procoxae and reaching the anterior border of prosternum (Bechyné 1951). The peculiar new genus also prompted the author to erect a new tribe to accommodate it, the Caryonodini Bechyné 1951. This new tribe was proposed to have affinities with the Iphimeites of previous classifications, including, among other, large New World genera such as *Antitypona* Weise 1921, *Brachypnoea* Gistel 1848, or *Spintherophyta* Lefèvre 1875, now part of Eumolpini Hope 1840 (Bouchard et al. 2011). However, this particular monotypic group has retained its individuality until today (Seeno and Wilcox 1982; Bouchard et al. 2011). The original generic description was accompanied with the report of two species from Bolivia and one from the State of Amazonas in Brasil (Bechyné 1951), of which, one Bolivian species, *Caryonoda kuscheli* Bechyné 1951, was selected as the type species. Bechyné (1953) added two additional species to the genus, including a new species from Venezuela, and a new combination for *Noda tibialis* Lefèvre 1885, from Colombia, which was formalized in a subsequent publication (Bechyné 1954). The final addition to this genus was the description of a new species from the State of Pará in Brasil, which also included a nice illustration of the very characteristic prosternum of all the species in the genus (Bechyné and Springlová de Bechyné 1961). At present, the genus includes six South American species in total, and in this work, we will present the first description of a Central American species of *Caryonoda* found in Nicaragua, which will represent the first new formal record of the genus, as well as a new species for the region.

The same revisionary work that helped us recognize the presence of *Caryonoda* in Nicaragua also revealed several new species and some new generic records for the country that will be reported, commented, and illustrated here as well.

## Material and methods

The specimens reported in this study were mostly picked by hand or collected sweeping vegetation between 2008 and 2012 in a range of tropical dry and rainforest biomes, in coastal, low elevations, and the mountainous region in the northwest of Nicaragua. Specimens were dissected and dry mounted including the male genitalia and female spermatheca after non-destructive proteinase K digestion for subsequent DNA extraction, and they are part of the research collections of the authors (JGZC, CSIC, Spain; and MEL, León, Nicaragua). Specimens were examined and photographed with a Leica M80 stereomicroscope equipped with a Leica FC420 digital camera, and pictures of the genitalia were used as the basis for line drawings for future reference. Specimen photographs where obtained with focus stacking using CombineZP (Alan Hadley, https://combinezp.software.informer.com). Identifications were made by the leading author using all the available literature as well as comparisons with pictures of type specimens when possible, made available by several institutions, including the Museum of Comparative Zoology (Cambridge, MA, USA), the Muséum National d’Histoire Naturelle (Paris, France), and the Museo del Instituto de Zoología Agrícola (Maracay, Venezuela). Nomenclature used for the species description followed Lawrence et al. (2010).

## Results and discussion

### Description of a new species of Nicaraguan Eumolpinae

*Caryonoda funebris* n. sp.

(Figs. 1c, 2a, 3k)

**Fig. 1:**
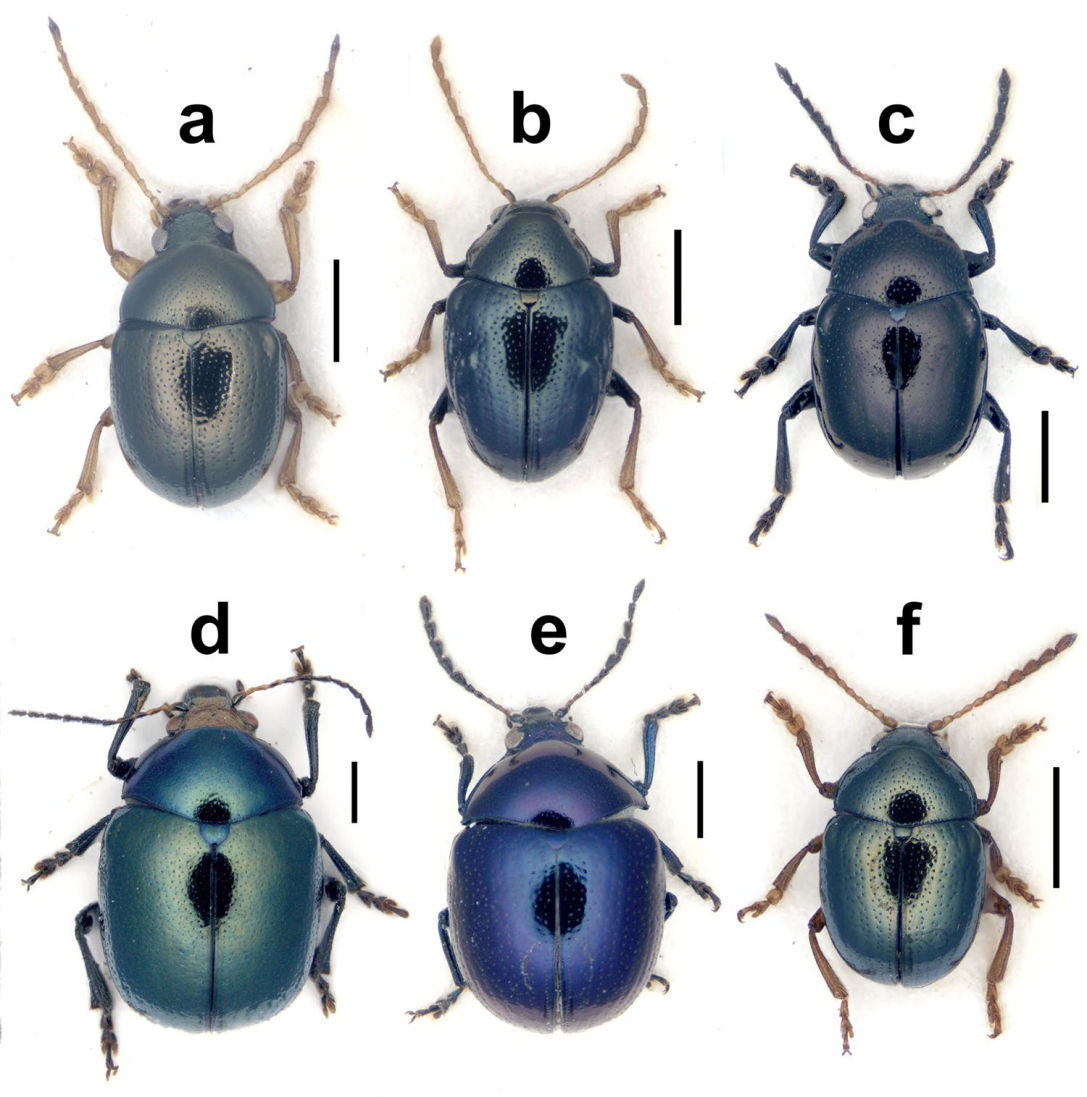
Dorsal views of some of the new records of Nicaraguan Eumolpinae in the tribe Eumolpini, including *Antitypona submetallica* (Jacoby) (**a**), *Brachypnoea modesta animatoria* (Bechyné) (**b**), *Caryonoda funebris* **n. sp. **(**c**), *Chrysodinopsis cupriceps* (Lefèvre) (**d**), *Spintherophyta ignita* (Lefèvre) (**e**), and *S. minuta* (Jacoby) (**f**). Scale bar = 1.0 mm

**Fig. 2:**
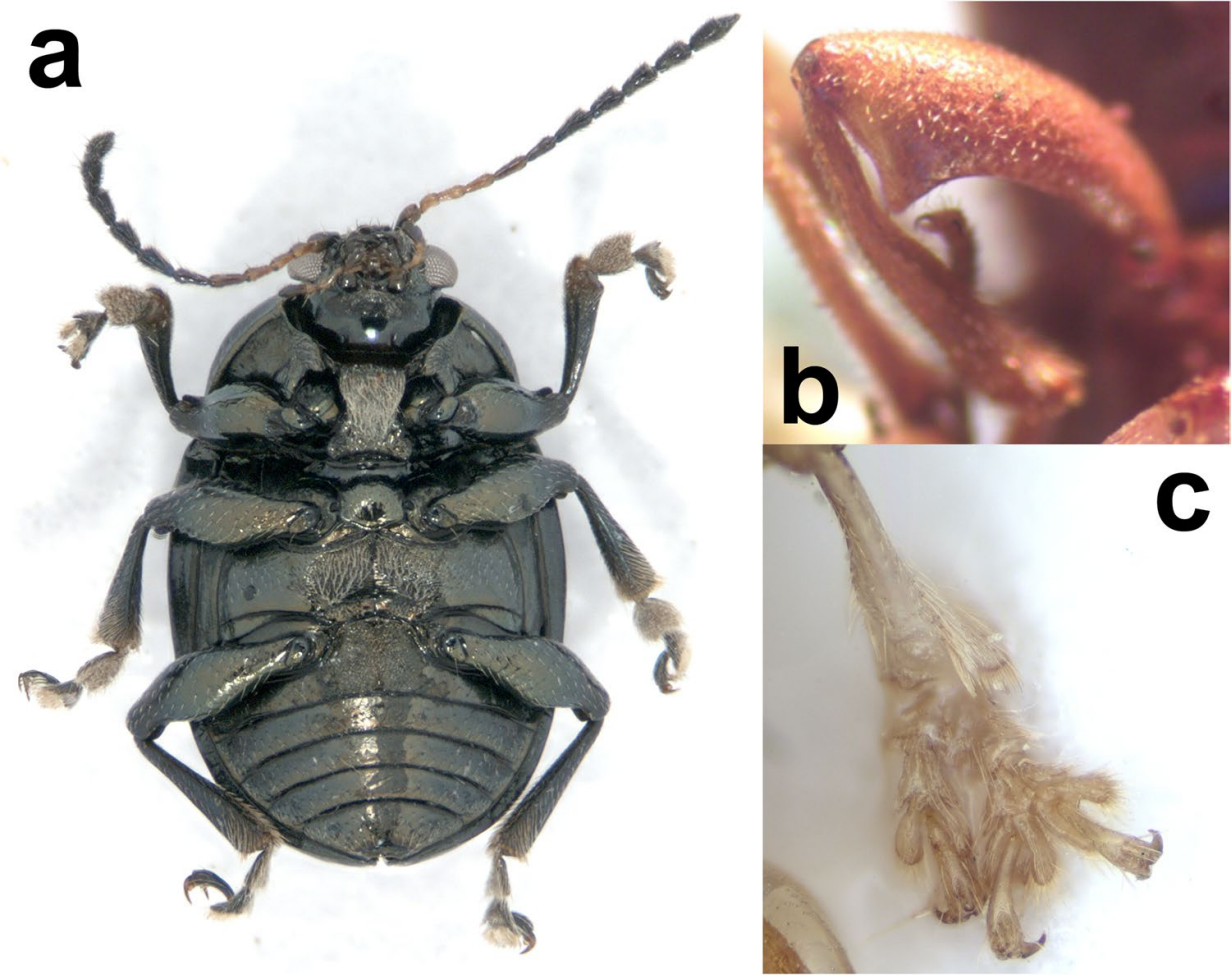
Ventral view of the holotype of *Caryonoda funebris* **n. sp.** where the apomorphic prosternal furrows of *Caryonoda* can be appreciated (**a**). Other panels showing details of other species discussed in the text, including the metafemoral tooth of the males of *Megascelis spinipes* Jacoby (**b**), and of an abnormal development in the right mesotarsus of *Tijucana vitticollis* (Jacoby) (**c**)

**Fig. 3:**
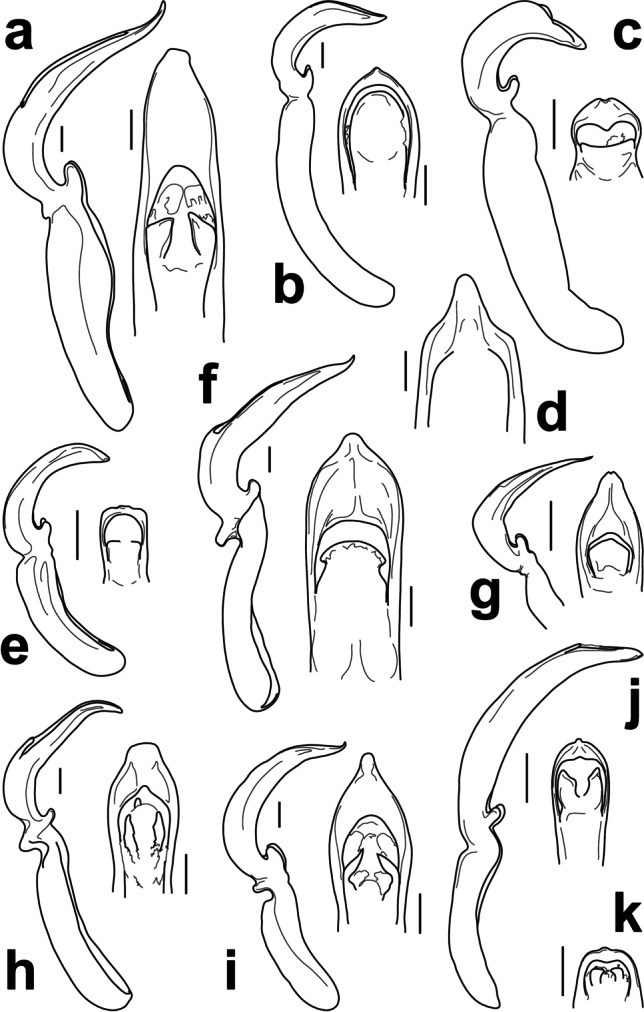
Lateral and dorsal apical views of the penis of *Colaspis jalapae* (Bechyné) (**a**), *Chrysodinopsis cupriceps* (Lefèvre) (**b**), *Antitypona submetallica* (Jacoby) (**c**), *Rhabdopterus uncotibialis* (Blake) (**d**), *Spintherophyta minuta* (Jacoby) (**e**), *Talurus tortonesei* Bechyné (**f**), *Typophorus subbrunneus* Jacoby (**g**), *Allocolaspis grandicollis* (Blake) (**h**), *Nodocolaspis femoralis* (Lefèvre) (**i**), *Brachypnoea modesta animatoria* (Bechyné) (**j**), and *Caryonoda funebris* **n. sp.** (**k**). Scale bar = 0.2 mm

urn:lsid:zoobank.org:act:0D19627C-5026-49BA-A9ED-477E24E8CC5B

Holotype (Figs. 1c, 2a): male (JGZC-1216), Nicaragua, Matagalpa, Selva Negra, 12°59′53.13″N 85°53′38.78″W, May 2010, Jean-Michel Maes leg., Holoype *Caryonoda funebris* n. sp. J. Gómez-Zurita det. [red label].

Body elongate elliptical, convex. Body jet black (Fig. 1c), with areas of metallic iridescence, including green reflections on antennal calli, clypeus and fine lateral margin and anterior angles of pronotum, and bluish reflections on scutellum, posterior angles of pronotum, humeral angles, and imperceptibly, depending on light incidence, on legs; basal maxillary palpomeres and four basal antennomeres ocher, with black dorsal metallic spot on scape; antennomeres 5–6 and distal maxillary palpomere dark brown, and remaining apical antennomeres black. Length: 3.9 mm; width: 2.4 mm.

Head broad, with interocular space more than half of head width; frontoclypeus with frons narrowly separated from clypeus by fine transverse furrow between narrow, transverse supraantennal calli; frons regularly convex, weakly depressed before calli, without traces of frontal suture, strongly microreticulate, matt, with fine shallow punctures smaller than intervals, except near eyes, and punctures with tiny translucent setae, slightly larger near eyes; clypeus small, longer than wide, bell-shaped, less than 1/3 as wide at base as frons between eyes, deflexed in apical half with moderate median emargination at apex, markedly microreticulate, punctured as frons in narrow basal area. Labrum slightly wider than base of clypeus, transverse, with round apical angles and anterior border slightly emarginate. Eyes convex, moderate, longer than wide, emarginate along inner border, more strongly convex and slightly stalked posteriorly. Last maxillary palpomere much longer and as wide as previous one, slightly cut at apex. Antennae filiform, reaching humeri, with antennomeres 7–11 longer (seventh longest and tenth shortest), slightly enlarged, finely rugose and more densely pubescent; antennomeres 5–6 subequal, slightly shorter than tenth, antennomeres 2–4 progressively shorter than fifth toward base of antennae, with pedicel slightly longer than wide, enlarged relative to third antennomere; scape longer than wide, dilated, about as long as seventh antennomere. Pronotum transverse (1.6 × as wide at base as long at middle) with sinuous sides, all angles acute and 1.8 × broader at base than at apex; markedly convex transversally and toward anterior angles; sides and angles finely margined, with margins finer and disappearing medially in slightly convex lobes on anterior and posterior borders; surface nearly smooth, glossy, with moderate, slightly aciculate punctures on disc, larger than punctures on frons and generally smaller than intervals, sparser near posterior border; punctures posteriorly at sides larger, tighter and more clearly aciculate; smooth intervals with scattered tiny punctures. Hypomera depressed on disc, smooth, unpunctured, with thickened margins parallel to border of pronotum, bent ventrally in inner part before large furrowed indentation of pronotum; inner bent area with abundant lanuginose whitish pubescence. Prosternum deeply furrowed at sides (Fig. 2a), prominent and flat medially, broader than transverse diameter of procoxae, laterally concave between procoxae and strongly widened posteriorly, with posterior border concave; surface irregular, glossy, with posteriorly recumbent dense lanuginose whitish pubescence in anterior half. Mesoventrite transverse, with broad short process, parallel-sided, nearly as wide as raised anterior part of prosternum, with posterior border obtuse, blunt; surface glossy, with fine punctures and posteriorly recumbent translucent setae. Mesepimera and mesanepisterna finely shagreened, unpunctured, glabrous. Metanepisterna elongate, wider anteriorly, finely shagreened, unpunctured and glabrous. Metaventrite markedly transverse, as long as prosternum at middle, with anterior border between mesocoxae slightly produced at obtuse angle and posterior border between metacoxae slightly concave; disc glossy, nearly unpunctured, with discrimen finely impressed, fine transverse scratches and dense lanuginose whitish pubescence except near posterior border; sides convex, relatively smooth, with sparse fine punctures and short, fine, posteriorly recumbent whitish setae. Scutellum slightly broader at base than long, arched at apex; surface glossy, with few sparse tiny punctures and microscopic setae. Elytra about 0.6 × as long as body, as wide at base as base of pronotum, sides weakly curved basally, widest in front of middle and gradually tapering toward regularly round apex, with borders finely explanate; markedly convex anteriorly, with strong humeri flanked internally by short longitudinal depression; surface smooth, glossier and with punctures smaller than on pronotum on disc, much smaller in apical half and laterally, forming regular longitudinal alignments in lateral and apical declivities, and irregular sparsely subgeminate series on disc. Epipleura wide at base, gradually narrowing posteriorly but not particularly thin and reaching sutural angle, slanted ventrally, entirely visible in lateral view; surface smooth, unpunctured, glabrous. Legs long, robust, with femora enlarged medially, particularly profemora, unarmed. Tibiae robust, shorter than corresponding femur gradually enlarged toward apex, more markedly in pro- and mesotibiae; pro- and mesotibiae feebly curved and metatibiae straight, with faint longitudinal keels and dense pale yellow setae ventrally at apex of meso- and metatibiae. Basitarsomeres of pro- and mesotarsi as long as tarsomeres 2–3 combined and notably wider, and basitarsomeres of metatarsi slightly shorter and narrower than corresponding tarsomeres 2–3; onychia as long or longer than basitarsomeres, clavate and slightly curved ventrally, carrying divaricate appendiculate claws. Pygidium short, weakly convex, with large median longitudinal furrow. Abdominal ventrites 1–4 strongly transverse, with posterior border concave; first ventrite as long as metaventrite at middle, with wide transverse subtrapezoidal process anteriorly; ventrites 2–4 subequal, half as long as first ventrite; fifth ventrite 1.5 × as long as fourth, strongly narrowed posteriorly, with biconvex apical emargination; surface of all ventrites smooth, glossy, with sparse fine punctures and posteriorly recumbent short fine translucent setae. Penis relatively broad and parallel, subtrapezoidal at apex with round angles and small median blunt projection; ostium occupying most of apex dorsally, covered by poorly sclerotized dorsal flap, longitudinally membranous at middle (Fig. 3k).

Diagnosis. *Caryonoda funebris* **n. sp.** is the largest representative of the genus so far, larger than 3.5 mm, when all the other species are smaller (the largest known species, with up to 3.0 mm, were *C. campanulicollis* Bechyné 1951 and *C. meridiana* Bechyné 1953). But apart from size, color is also an easy feature to distinguish the new species, since it is the only one known to have black dorsum with faint, nearly imperceptible metallic bluish or purplish sheen, when all the other species have black dorsum but with strong bronzy, greenish, bluish or purplish metallic shine on pronotum and elytra. The legs of *C. funebris* are also entirely black with faint bluish or purplish shine and in the other species they have at least apex of tibiae and tarsi testaceous. The punctation of pronotum and elytra of *C. funebris* is very fine and sparse, relatively homogenously distributed on pronotum and sparsely subgeminate on disc and aligned as single rows in apical declivity of elytra; punctation in other species is relatively strong, denser, both on pronotum and elytra, in most species leaving wide unpunctured areas basally on pronotum.

*Derivatio nominis.* The species name is an adjective (f.) derived from the Latin *fūnŭs, -ĕris* (neut.), meaning funeral, or the ceremony to honor deceased people. The name is used in reference to the black color of the species compared to the festive metallic colors of other species. Black is used as a somber sign of mourning in Western cultures, possibly inherited from the classical Roman tradition and the use of the so-called *toga pulla*, the dark-colored toga, in the funerals of renowned people.

Distribution. The genus *Caryonoda* was currently known from localities south of the Isthmus of Panama, including *C. meridiana* in Venezuela, *C. tibialis* (Lefèvre 1885) in Colombia, *C. campanulicollis* and *C. kuscheli* Bechyné 1951 in Bolivia (the latter also in Peru), and *C. bisinuata* Bechyné and Bechyné 1961 and *C. pohli* Bechyné 1951 in Brasil. The presence of *C. funebris* **n. sp.** in Nicaragua represents the first record of the genus in Central America. The only known specimen is from the cloud forest of Matagalpa, consistent with the previous records of the genus in humid biomes east of the Andean cordillera.

### New genera and species records of Nicaraguan Eumolpinae

The confirmed presence of *Caryonoda funebris* n. sp. in Nicaragua represents the first formal record for this genus in Nicaragua, but also in Central America. But in the course of our work in Nicaragua, we found several other new genera and species records, in this case for the country. The list below reports these findings with commentaries about the species, their identification, and their distributions, and provides illustrations to help interpreting our findings in future studies.

***Allocolaspis grandicollis*** **(Blake,** 1976a **)**

(Figs. 3h, 4a)

**Fig. 4:**
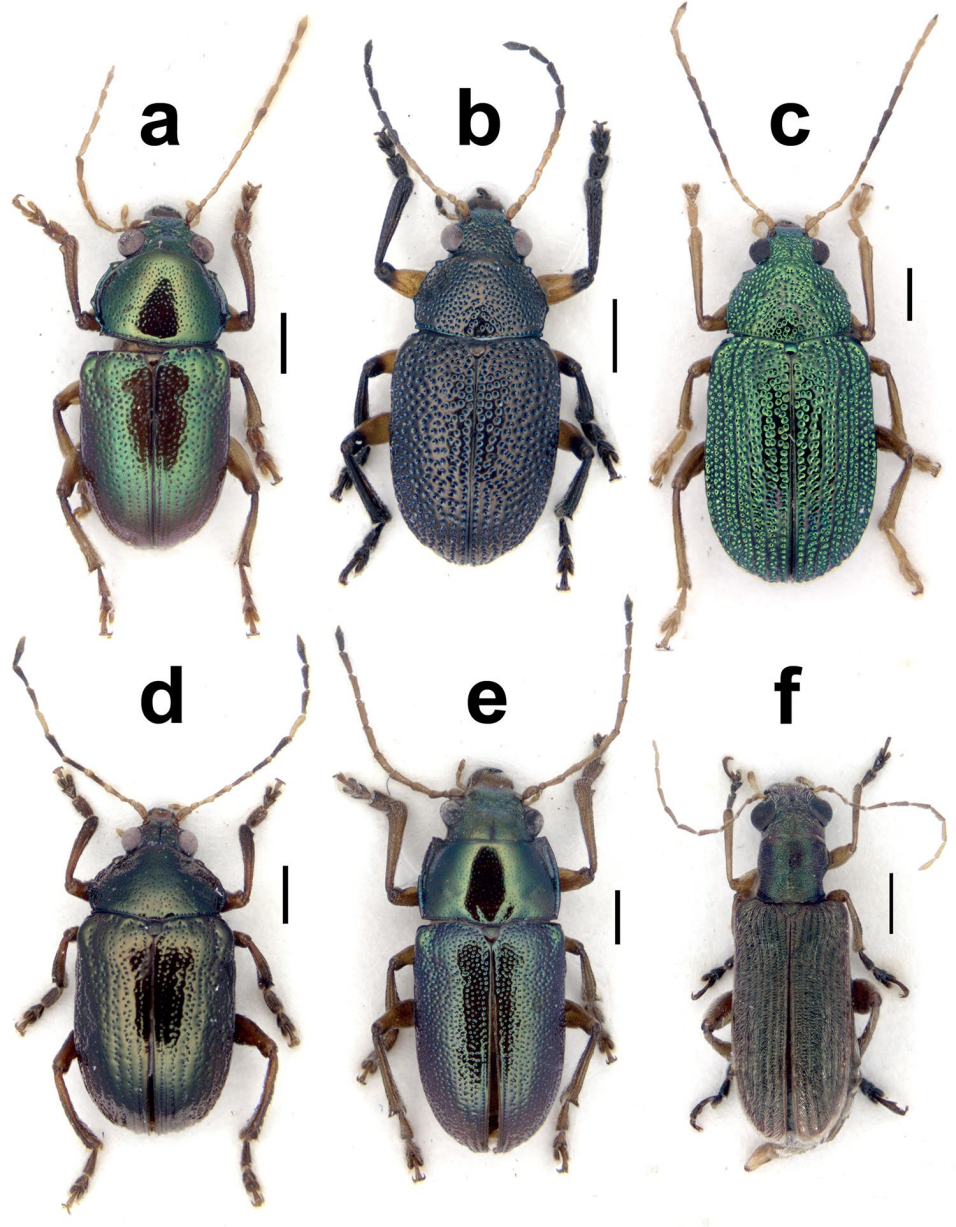
Dorsal views of some of the new records of Nicaraguan Eumolpinae in the tribes Eumolpini and Megascelini, including *Allocolaspis grandicollis* (Blake) (**a**), *Nodocolaspis femoralis* (Lefèvre) (**b**), *Colaspis jalapae* (Bechyné) (**c**), *Rhabdopterus uncotibialis* (Blake) (**d**), *Talurus tortonesei* Bechyné (**e**), and *Megascelis spinipes* Jacoby (**f**). Scale bar = 1.0 mm

New species record: **JGZC:** (1) male (JGZC-1236), Rivas, Cárdenas, Finca Sierra Serena, 11.234449 -85.5539179, 66 m, 14 July 2010, J.-M. Maes leg., *Allocolaspis grandicollis* (Blake) J. Gómez-Zurita det. 2022. **MEL:** (1) 1 ex., #232, Región Autónoma de la Costa Caribe Norte, Reserva Biosfera BOSAWAS, Cerro Saslaya, 13.75054 -84.97857, 250–300 m, 6–10 May 2011, B. Hernández leg.; (2) 2 exx., #152, Región Autónoma de la Costa Caribe Norte, 8 km E Bonanza, Centro de Información y Comunicación Regional para el Trópico Húmedo (CICABO), 14.013335 -84.525334, 21 August 2000, sobre cacao, Y. Dixon leg., *Allocolaspis sp.* W. Flowers det. 2020; (3) 1 ex., #270, *idem*, 8 September 2000, J. Flores leg.; (4) 1 ex., *idem*, 12 December 2000, sobre aguacate, Y. Dixon leg.; (5) 1 ex., Río San Juan, Los Guatuzos, Río Papaturro, 11.032255 -85.053492, 40 m, 17–24 March 2000, J.-M. Maes, J. Sunyer & B. Hernández leg.; (6) 1 ex., Rivas, Mata de Caña, 11.554471 -85.968130, 110 m, 31 July–1 August 2002, en bosque ripario, B. Hernández leg.

*Allocolaspis* Bechyné 1950a, as currently defined, is a genus with species distributed in northern South America, Central America south from Guatemala, and the Antilles (Bechyné 1953; Flowers 1996). Fourteen species were reported from the region of interest, with one of them endemic from Nicaragua, *A. belti* (Jacoby 1881), and the only record of the genus in the country. The current species, originally described from Panama, and confirmed studying the penis (Fig. 3h) of the only specimen collected in Nicaragua (Fig. 4a), would be the second registered species of *Allocolaspis* in this country.

***Antitypona submetallica*** **(Jacoby,** 1890 **)**

(Figs. 1a, 3c)

New species record: **JGZC:** male (JGZC-1211), Granada, Reserva Silvestre Privada Domitila, May 2010, J.-M. Maes leg., *Antitypona submetallica* (Jac.) J. Gómez-Zurita det. 2022.

There is some confusion about the limits between the genera *Antitypona* and *Lamprosphaerus*, two genera of typically small, roundish, and metallic beetles with a facies similar to *Spintherophyta* and *Brachypnoea*, but without clavate antennae. Both genera can be somewhat distinguished by the shape of hypomera, longitudinally indented in *Lamprosphaerus* and relatively convex in *Antitypona* (Weise 1921), and also by the degree of inclination of epipleura, horizontal in *Lamprosphaerus* and slanted, visible laterally in *Antitypona* (Flowers 1996). A single male specimen from Nicaragua (nearly 3 mm long) is very similar to the female type specimen of *A. submetallica* (Jacoby, 1890) except in the marked dorsal metallic green color and the antennae more slender and nearly entirely pale (except last antennomere), but the latter also show the enlarged pedicel and short and fine antennomeres 3–4 as in the type and the same fine punctation on head and pronotum over microreticulated background (Fig. 1a). This male is tentatively classified as Jacoby’s species, also taking into account that all the other species of *Antitypona*, except the characteristic *A. apicalis* (> 3.5 mm), are much smaller (< 2.6 mm), but the penis is figured to allow for future assessment of our interpretation (Fig. 3c). The species is known from Costa Rica and Panama (Flowers 1996), and the Nicaraguan record would extend the range of the species to the north.

***Brachypnoea modesta animatoria*** **(Bechyné,** 1953 **)**

(Figs. 1b, 3j, 5a)

**Fig. 5:**
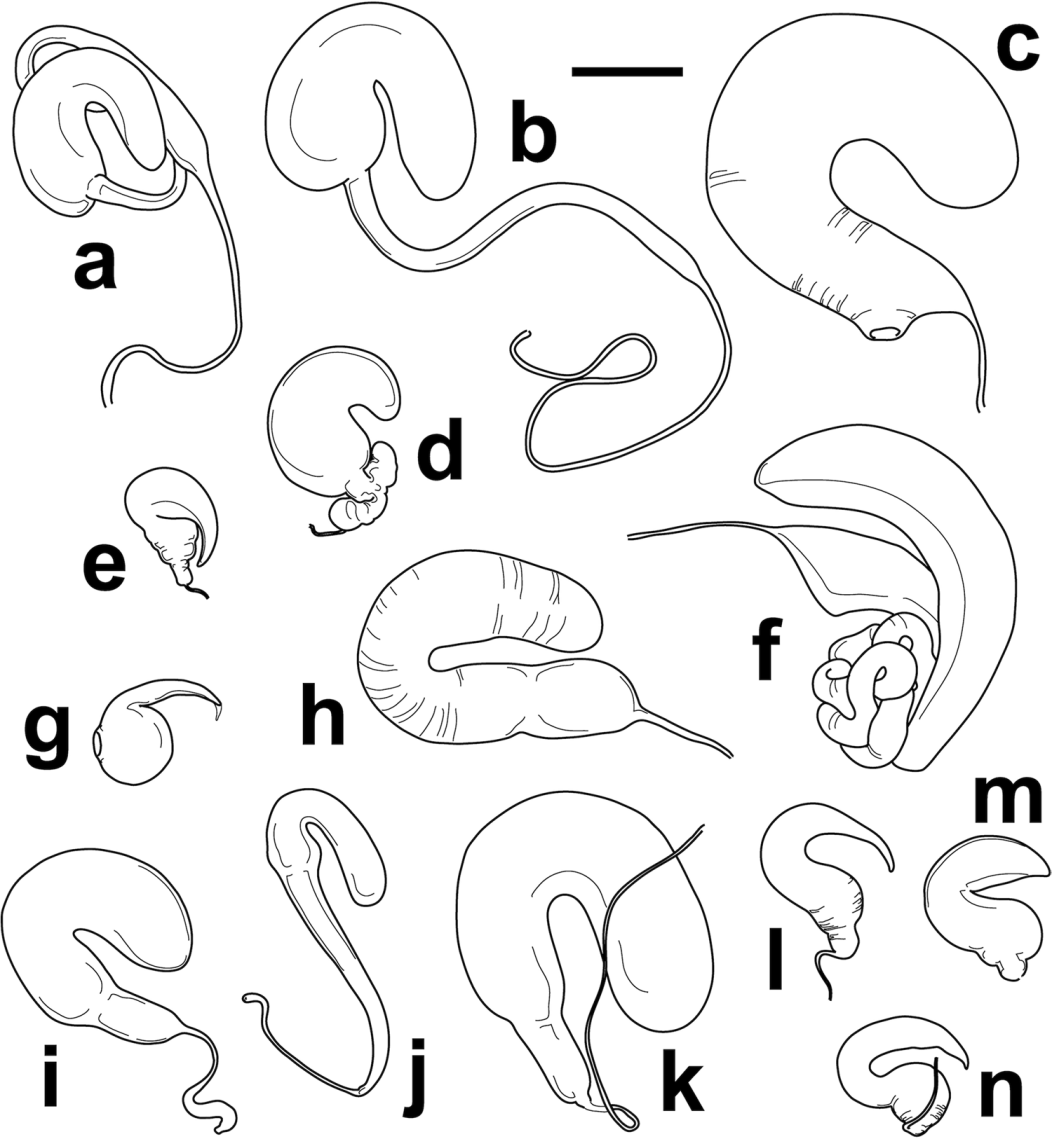
Spermathecae of *Brachypnoea modesta animatoria* (Bechyné) (**a**), *Chrysodinopsis cupriceps* (Lefèvre) (**b**), *Colaspis jalapae* (Bechyné) (**c**), *Megascelis spinipes* Jacoby (**d**), *Metachroma panamense* Blake (**e**), *Nodocolaspis femoralis* (Lefèvre) (**f**), *Paria nigritarsis* Jacoby (**g**), *Rhabdopterus uncotibialis* (Blake) (**h**), *Spintherophyta ignita* (Lefèvre) (**i**), *S. minuta* (Jacoby) (**j**), *Talurus tortonesei* Bechyné (**k**), *Typophorus apicicornis* Jacoby (**l**), *T. cyanipennis* Lefèvre (**m**), and *T. subbrunneus* Jacoby (**n**). Scale bar = 0.2 mm

New species record: **JGZC:** 2 females (JGZC-1299 and 1313) and 2 males (JGZC-1314 and 1315), Rivas, Sapoa, Finca Guadalupe, 11.179983 -85.6807925, 15 September 2009, J.-M. Maes leg., *Brachypnoea modesta animatoria* (Bech.) J. Gómez-Zurita det. 2022.

*Brachypnoea* Gistel, 1848 is one of the most diverse genera of Central American Eumolpinae, also in Nicaragua, where five species were reported so far (Maes and Staines 1991). The new specimens of *Brachypnoea* collected in Nicaragua belong to an informal group of species that could be characterized by small size (around 3.0 mm or less), fulvous antennae and legs, as well as the presence of tubercles and costae on the elytra of females, among other traits. A particular feature of these specimens is that they have fulvous tibiae and tarsi, but dark femora, which is a combination not that commonly seen in *Brachypnoea*. *B. thoracica* (Jacoby, 1881) and *B. opacicollis* (Jacoby, 1890) from Mexico show bicolor legs, but they have the antennae with apical antennomeres dark and females lack basal tubercles on elytra, and they have differences in texture and punctation of pronotum as compared to the Nicaraguan specimens (*B. thoracica* is much larger too). *B. boucardi* (Jacoby, 1878) from Guatemala, another species with pale tibiae and tarsi, is not that different to the insects from Nicaragua, but the species was described as having dark apical antennomeres and the type material in the Museum of Comparative Zoology, a male, possibly the holotype, shows differences in the punctation of pronotum and elytra, which also have faint apical costae, missing in the Nicaraguan males. Moreover, Bechyné (1955) subordinated *B. boucardi* to *B. lefevrei* (Jacoby, 1878), which in case that this was a sound assessment, it would imply that the females lack tubercles on elytra, unlike the specimens from Nicaragua. *B. fulvicornis* (Jacoby, 1890) from Mexico occasionally has darker femora, but this species has dull pronotum with very fine punctation, compared to the Nicaraguan specimens. Something very similar could be said for *B. parvula* (Jacoby, 1890) from Panama, typically having bicolor legs, but fine, dull sculpture on pronotum. However, *B. parvula* belongs to an unstable taxonomic complex that has experienced some changes in rank and combination, including the incorporation of traits that fit the Nicaraguan specimens. For example, Bechyné (1953) described *B. parvula animatoria* from Costa Rica and Guatemala, precisely highlighting the stronger punctation on pronotum of these specimens compared to typical *B. parvula*. Later, Bechyné (1955) subordinated *B. parvula* to *B. modesta* (Lefèvre, 1878), a species characterized by the presence of a tubercle posterior to the prebasal depression of elytra, present in the Nicaraguan specimens (Fig. 1b). Based on these considerations, we are inclined to agree with the taxonomic solution adopted by Flowers (1996), considering the combination *B. modesta animatoria*, where we refer our Nicaraguan specimens, thus adding an intermediate locality to the current disjunct distribution between Guatemala and Costa Rica. With the aim to assist future reanalysis of these taxa and how they relate to each other, we provide with illustrations of the male genitalia (Fig. 3j) and spermatheca (Fig. 5a).

***Chrysodinopsis cupriceps*** **(Lefèvre,** 1877 **)** **n. comb.**

*Chrysodina cupriceps* Lefèvre, 1877

(Figs. 1d, 3b, 5b)

New species record: **JGZC:** (1) female (JGZC-1375), Estelí, Reserva Natrual Miraflor, Finca Beylla Vista, Mirador Los Cerritos, 13.263 -86.2293 [118], July 2010, A. Del Socorro & B. Nimia leg., *Chrysodinopsis cupriceps* (Lef.) J. Gómez-Zurita det. 2022; (2) female (JGZC-1333), Estelí, Area Protegida Miraflor-Moropotente, July 2010, on *Acanthospermum hispidum*, A. del Socorro & B. Nimia leg., *Chrysodinopsis cupriceps* (Lef.) J. Gómez-Zurita det. 2022; (3) female (JGZC-1753), Estelí, Reserva Natural Miraflor, Comunidad Isiqui, 13.113206 -862,244,199, August 2010, on *Astronium graveolens*, A. del Socorro & B. Nimia leg., *Chrysodinopsis cupriceps* (Lef.) J. Gómez-Zurita det. 2022; (4) female (JGZC-1757), Estelí, Reserva Natural Miraflor, Comunidad Isiqui, 13.106656 -86.2320785, August 2010, on *Datura stramonium*, A. del Socorro & B. Nimia leg., *Chrysodinopsis cupriceps* (Lef.) J. Gómez-Zurita det. 2022; (5) female (JGZC-1760), Estelí, Reserva Natural Miraflor, Comunidad Isiqui, 13.106656 -86.2320785, August 2010, on *Datura stramonium*, A. del Socorro & B. Nimia leg., *Chrysodinopsis cupriceps* (Lef.) J. Gómez-Zurita det. 2022; (6) female (JGZC-1763), Estelí, Reserva Natural Miraflor, Comunidad Isiqui, 13.106656 -86.2320785, August 2010, on *Milleria quinqueflora*, A. del Socorro & B. Nimia leg., *Chrysodinopsis cupriceps* (Lef.) J. Gómez-Zurita det. 2022; (7) female (JGZC-1765), Estelí, Reserva Natural Miraflor, Comunidad Isiqui, August 2010, A. del Socorro & B. Nimia leg., *Chrysodinopsis cupriceps* (Lef.) J. Gómez-Zurita det. 2022; (8) male (JGZC-1230), Rivas, Finca Guadalupe, 11.179983 -85.6807925, 290 m, 10–12 July 2010, J.-M. Maes leg., *Chrysodinopsis cupriceps* (Lef.) J. Gómez-Zurita det. 2022.

Jacoby (1890) reflected on *Chrysodina cupriceps* and suggested that it may be better placed with *Noda curtula*, and both in his *Noda* (currently, *Brachypnoea*), based on their morphological similarities. Bechyné (1950b) created the monotypic genus *Chrysodinopsis* for *N. curtula* based on differences in the shape of elytra and epipleura relative to typical *Brachypnoea,* and later transferred *N. basalis* to this genus too (Bechyné 1957), while retaining *C. cupriceps* in *Chrysodina* (Bechyné 1953). Flowers (1996) respected the limits to *Chrysodinopsis* established by Bechyné (1957) and transferred *C. cupriceps* to *Brachypnoea*. Van Roie et al. (2019) reported *C. cupriceps* from El Salvador, placing it in the genus *Chrysodinopsis*, but without justification. After comparing *C. cupriceps* with *C. curtula*, we agree with Jacoby’s (1890) original assessment on the strong morphological similarity between both species, including slightly flattened apex of elytra in the only male examined. While the validity of *Chrysodinopsis* remains unchallenged, both species should be placed in the same genus based on their morphological proximity; thus, we formalize the new combination here. Previously reported from El Salvador, Guatemala, Honduras, and Mexico (Flowers 1996; Van Roie et al. 2019), the presence of *C. cupriceps* in Nicaragua would extent the range of the species considerably to the south. Nicaraguan specimens range from cupreous to metallic green, blue, purple, and almost black, sometimes with slight differences in the hue of pronotum and elytra, but the head is always of a different color compared to the rest of the body (Fig. 1d). We illustrate the penis and the spermatheca of the species for future comparisons (Figs. 3b and 5b).

***Colaspis jalapae*** **(Bechyné,** 1950c **)**

(Figs. 3a, 4c, 5c)

New species record: **JGZC:** (1) male (JGZC-1790), Estelí, Reserva Natural Miraflor, 13°13′16″N 86°14′56″W, 1389 m, 10 September 2011, on *Solanum myriacanthum*, J.-M. Maes leg., *Colaspis jalapae* (Bech.) J. Gómez-Zurita det. 2022; (2) male (JGZC-1795), Estelí, Reserva Natural Miraflor, 13°15′19″N 85°15′18″W, 1300 m, 10 September 2011, on *Salvia comayaguana*, J.-M. Maes leg., *Colaspis jalapae* (Bech.) J. Gómez-Zurita det. 2022; (3) female (JGZC-2636), Estelí, Reserva Natural Miraflor, La Neblina del Bosque (jardín), 13°14′45″N 86°14′57″W, 1350 m, 28 January 2012, A. Cardoso, G. De la Cadena & A. Papadopoulou leg., *Colaspis jalapae* (Bech.) J. Gómez-Zurita det. 2022; (4) female (JGZC-1411), Jinotega, nr. El Mojón, Reserva El Jaguar, 13.237929 -86.052792, 1314 m, 20–21 November 2010, J.-M. Maes leg., *Colaspis jalapae* (Bech.) J. Gómez-Zurita det. 2022; (5) male (JGZC-0143), Matagalpa, Selva Negra, 12°59′53.13″N 85°53′38.78″W, 27 October 2001, P. Jolivet leg., *Colaspis jalapae* (Bech.) J. Gómez-Zurita det. 2022; (6) male (JGZC-1319), *idem*, October 2009, J.-M. Maes leg.; male (JGZC-1212), *idem*, May 2010, J.-M. Maes leg.; (7) male (JGZC-1407), *idem*, November 2010, J.-M. Maes leg.; female (JGZC-1227), Región Autónoma Atlántico Norte, 7 km N Waslala, Cañón Los Martínez, 900 m, June 2009, Fr. LePont leg., *Colaspis jalapae* (Bech.) J. Gómez-Zurita det. 2022. **MEL:** (1) 20 exx., #130, Estelí, Paisaje Natural Protegido Miraflores-Moropotente, 13.245704 -86.254129, 1434 m, 26–27 October 2001, J.-M. Maes & B. Téllez leg.; (2) 4 exx., Jinotega, road Matagalpa-Jinotega, km 147.5, 13.044723 -85.934450, 1500 m, 20 November 1994, J.-M. Maes, J. Téllez & J. Hernández leg., *Colaspis prasina* C.L. Staines det. 1996; (3) 2 exx., #129, *idem*, 3 December 1994, *Colaspis sanjoseana* R. Westerduijn det. 2020; (4) 2 exx., Jinotega, El Jaguar, 13.237978 -85.052655, 1346 m, 25 October 2003, J.-M. Maes leg.; (5) 3 exx., Matagalpa, Fuente Pura, 13.010834 -85.920830, 1550 m, 20 March 1994, J.-M. Maes & A. De La Fuente leg.; (6) 1 ex., #60, *idem*, 10 April 1994, *Colaspis sanjoseana* R. Westerduijn det. 2020; (7) 1 ex., *idem*, 12 June 1994, J.-M. Maes, J. Téllez & J. Hernández leg.; (8) 3 exx., #61, *idem*, 26 June 1994; (9) 3 exx., *idem*, 21 July 1994; (10) 3 exx., *idem*, 16 August 1994, J.-M. Maes & J. Hernández leg.; (11) 7 exx., *idem*, 4 September 1994, J.-M. Maes leg.; (12) 8 exx., *idem*, 5 September 1994; (13) 1 ex., *idem*, 30 April 1995, J.-M. Maes, J. Téllez, J. Puig, V. Hellebuyck & J. Hernández leg.; (14) 3 exx., *idem*, March 1997, J.-M. Maes, J. Téllez & J. Prena leg.; (15) 1 ex., *idem*, 24–25 April 1999, J.-M. Maes & B. Hernández leg.

The genus *Colaspis* Fabricius, 1801 (the *Maecolaspis* Bechyné, 1950b of most Neotropical catalogs; also in Maes and Staines 1991) is definitely one of the most diverse genera of American Eumolpinae. The genus is well represented in Nicaragua, where at least seven species were reported (Maes and Staines 1991). Here, we report a new species of *Colaspis* from Nicaragua that belongs in the bulk of very similar species occurring throughout America, and highly diverse in Central America, typically characterized by bright metallic green dorsum and fulvous legs and antennae, and very difficult taxonomically, since most species are very similar externally (Bechyné 1950c). *C. jalapae* was described from Veracruz (Mexico) and has not been reported anywhere else; thus, the presence of the species in Nicaragua would represent a significant expansion of the known range. Given the taxonomic difficulties of this group, there is a chance that the identification of Nicaraguan specimens is wrong, but these large (6.4–7.2 mm) and brightly colored specimens, even though they are not as big as reported in the original description (i.e., 8 mm), share the most important traits with the Mexican species, as to leave little doubt about their identification or, at the very least, their taxonomic proximity. Some of these traits include the darkened interstitial antennomeres and also the abrupt median enlargement of male metatibiae (Bechyné 1950c). One female specimen as well as the male genitalia and the spermatheca are illustrated for future reference (Figs. 3a, 4c, 5c).

***Megascelis spinipes*** **(Jacoby, 1888)**

(Figs. 2b, 4f, 5d)

New species record: **JGZC:** (1) female (JGZC-1490), Estelí, Reserva Natural Miraflor, Finca Las Flores, 13.189271 -86.325221, 926 m, March 2010, A. del Socorro & B. Nimia leg., *Megascelis spinipes* Jac. J. Gómez-Zurita det. 2022; (2) female (JGZC-1510), Granada, Reserva Silvestre Privada Domitila, 7 November 2010, J.-M. Maes leg., *Megascelis spinipes* Jac. J. Gómez-Zurita det. 2022. **MEL:** (1) male, Carazo, Reserva Chacocente, 11–13 November 1992, bosque tropical seco, Maes, Martínez & López leg., *Megascelis sp.* C. L. Staines det. 1993; (2) 2 exx., Granada, R. S. P. Domitila, UTM 16P 0,558,750—1,334,939, 230 m, 25 November 2003, J.-M. Maes leg.; (3) 1 ex., *idem*, 14–18 April 2004; (4) 1 ex., *idem*, May 2005; (5) 1 ex., *idem*, 12–15 December 2006; (6) 1 ex., Granada, Volcán Mombacho, Finca El Progreso, 30 May 1999, trampa Malaise en cafetal con tratamiento químico, J.-M. Maes leg.; (7) 1 ex., Managua, La Chinampa, UTM 16P 0,558,750—1,334,939, 230 m, 26–29 September 2001, J.-M. Maes leg.

This is a member of another large, taxonomically complicated group of mostly Neotropical Eumolpinae (Lacordaire 1845), which is also relatively diverse in Nicaragua, where up to four species have been reported so far (Maes and Staines 1991). This would be the first Nicaraguan report of *M. spinipes*, a species cited from Guatemala and southern Mexico (Flowers 1996), and we are confident on our identification based on the male, displaying a pronounced tooth in metafemora (Fig. 2b), and two female specimens (Fig. 4f) compared with one of the species syntypes in the Museum of Comparative Zoology (Harvard, USA). There are several distinguishing traits for the species, apart from the notorious metafemoral modification in males, including the dark, relatively dull green color of dorsum with a broad ocher lateral stripe on elytra, tight transverse punctures and dense golden pubescence of elytra, or the finely transversely rugose texture of pronotum among others. We also illustrate the spermatheca of the species (Fig. 5d), which would have a much larger range than originally assumed.

***Metachroma panamense*** **(Blake,** 1970 **)**

(Figs. 5e, 6a)

**Fig. 6:**
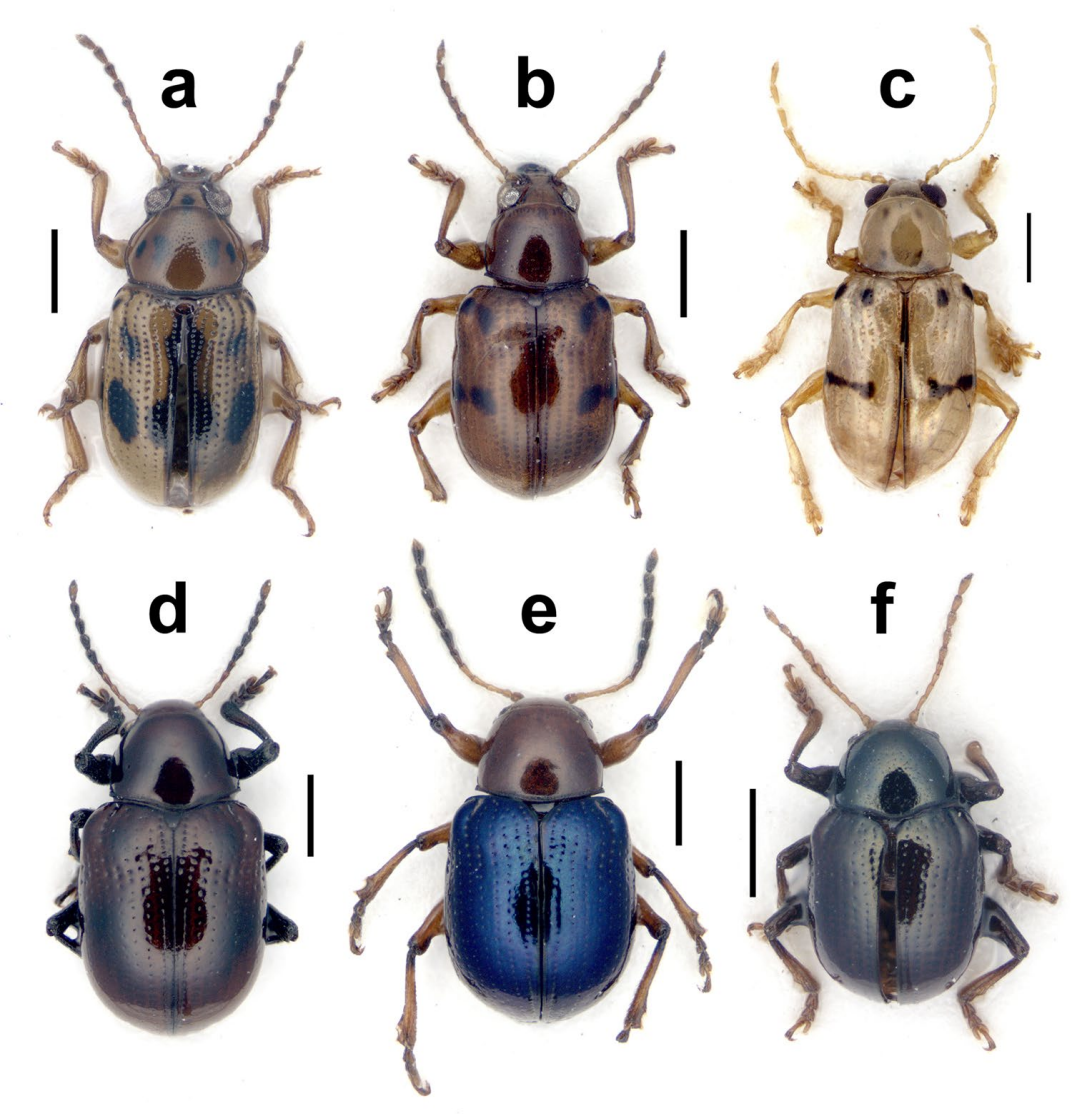
Dorsal views of the new records of Nicaraguan Eumolpinae in the tribe Typophorini, including *Metachroma panamense* Blake (**a**), *Paria nigritarsis* Jacoby (**b**), *Tijucana vitticollis* (Jacoby) (**c**), *Typophorus apicicornis* Jacoby (**d**), *T. cyanipennis* Lefèvre (**e**), and *T. subbrunneus* Jacoby (**f**). Scale bar = 1.0 mm

New species record: **JGZC:** female (JGZC-2594), León, Salinas Grandes, Reserva Natural Isla de Juan Venado, 12.27217 -86.88130, 36 m, 24 January 2012, J. Gómez-Zurita, A. Cardoso, G. De la Cadena & A. Papadopoulou leg., *Metachroma clarkei* Blake J. Gómez-Zurita det. 2022. **MEL:** 2 exx., #70, Carazo, Chacocente, 11.534389 -86.188843, 5 m, 11–13 September 1992, trampa de luz en bosque tropical seco, J.-M. Maes, A. Martínez & R. López leg., *Metachroma sp.* C. L. Staines det. 1993.

*Metachroma* Chevrolat, 1836 was comprehensively revised by Blake (1970) and she raised the number of species found in Central America from twelve up to 25 species, most of them from Mexico, and three of them reported from Nicaragua already (Maes and Staines 1991). *M. panamense* is a species originally described from Panama, as the name suggests, and characterized by the presence of four dark spots on the pronotum, vittate elytra, and differences in punctation and sculpture on pronotum and elytra compared to other species with spotted pronotum, as well as the lack of convex lateral intervals in female elytra (Blake 1970). The specimen from Nicaragua fits the description of *M. panamense* in most respects, including the relatively short inner appendix of bifid claws, the presence of median dark spots on disc of elytra close (actually fused) to suture, pale margins of pronotum and scutellum, and a relatively small size (length of female = 4.1 mm). However, the specimen also shares some traits with *M. clarkei* Blake, 1970, a larger Mexican species described from Yucatan, but very similar externally to *M. panamense*, although they present some distinctive traits which are present in the Nicaraguan specimen. These would include darkened apical antennomeres, presence of subhumeral and median dark spots fused to lateral margin of elytra, the prominent spots on elytra with sutural stripe enlarged behind scutellum, or the relatively strong punctation on elytra, attenuated apically, but clearly distinct on disc. Both species apparently have very similar male genitalia, based on Blake’s (1970) illustrations, and it is entirely possible that they are indeed conspecific. However, until the genus is revised again with more data, we are inclined to consider the Nicaraguan record akin to the Panamanian, rather than the Mexican taxon. The specimen (Fig. 6a) and the spermatheca (Fig. 5e) are figured to assist future taxonomic reassessment.

***Nodocolaspis femoralis*** **(Lefèvre,** 1878 **)**

(Figs. 3i, 4b, 5f)

New species record: **JGZC:** (1) male (JGZC-2640), León, La Paz Centro, rd to Geotérmica, between road and swamp, lagood after Loma de Chistata, 12.43437 -86.59300, 66 m, 1 February 2012, A. Cardoso, G. De la Cadena, J.-M. Maes & A. Papadopoulou leg., *Colaspis balyi* Jac. J. Gómez-Zurita det. 2022; (2) male (JGZC-1954), Managua, Montelimar, Reserva Natura, 26 February 2011, on *Cissus verticillata*, J.-M. Maes leg., *Colaspis balyi* Jac. J. Gómez-Zurita det. 2022; (3) female (JGZC-2592), Managua, Montelimar, Reserva Natura, 11.87659 -86.50341, 125 m, 22 January 2012, J. Gómez-Zurita, A. Cardoso, G. De la Cadena & A. Papadopoulou leg., *Colaspis balyi* Jac. J. Gómez-Zurita det. 2022. **MEL:** (1) 1 ex., #277, Región Autónoma de la Costa Caribe Norte, 8 km E Bonanza, Centro de Información y Comunicación Regional para el Trópico Húmedo (CICABO), 14.013335 -84.525334, 100 m, 17–20 November 2000, J.-M. Maes & B. Hernández leg., *Nodocolaspis femoralis* M. Geiser det. 2020; (2) 1 ex., #278, Región Autónoma de la Costa Caribe Norte, Wani, 13.697231 -84.850277, 100 m, November 1995, J.-M. Maes & J. Hernández leg., *Nodocolaspis femoralis* M. Geiser det. 2020; (3) 1 ex., #214, Río San Juan, Los Guatuzos, Río Papaturro, 11.032255 -85.053492, 40 m, 17–24 March 2000, J. Sunyer & B. Hernández leg., *Nodocolaspis femoralis* M. Geiser det. 2020.

This species was originally described in the genus *Colaspis* and it is reminiscent of the informal group of *C. melancholica*, as defined by Blake (1976b), characterized by species of nearly black dorsum with traces of metallic shine and strong, dense dorsal punctation. However, it was transferred to the genus *Nodocolaspis* Bechyné, 1949 by Bechyné (1953), who recognized the synonymy with *Colaspis rufofemorata* Lefèvre, 1884 and the proximity of the species to *N. tarsata* (Lefèvre, 1882), the latter clearly in the genus *Nodocolapis* according to this author, based on the shape of prosternum, antennae and male protarsi. Subsequent authors, such as Flowers (1996), disregarded this taxonomic decision although expressed doubts about the correct placement of *Colaspis femoralis*. We agree with the interpretation by Bechyné (1953) and retain his classification to emphasize the position of this species outside of the genus *Colaspis*. The species is known from Colombia, Venezuela, Panama, and the State of Amazonas in Brasil (Lefèvre 1878, 1884; Jacoby 1890; Bechyné 1955, 1958), and it shows some variability in the coloration of legs (Bechyné 1953), including red femora and tibiae, red femora and black tibiae, or red femora with black apex and black tibiae, the latter corresponding to the type of *C. rufofemorata*, the variant we found in Nicaragua (Fig. 4b) and also reported by Jacoby (1890) from Panama. If the conspecificity of specimens from the Amazonas to Nicaragua were confirmed, this would be one of the species of American Eumolpinae with a larger range across the Isthmus of Panama. We illustrate the penis (Fig. 3i) and spermatheca (Fig. 5f) of this species for the first time.

***Paria nigritarsis*** **(Jacoby, 1882)**

(Figs. 5g, 6b)

New genus and species record: **JGZC:** female (JGZC-2596), Rivas, Cárdenas, Finca Isla Vista, 11.23345 -85.55123, 56 m, 8 February 2012, A. Cardoso, G. De la Cadena & A. Papadopoulou leg., *Paria nigritarsis* Jac. J. Gómez-Zurita det. 2022.

*Paria* LeConte, 1858 is a genus relatively diverse in North America and with some twenty species in the Neotropics (Bechyné 1953). In Central America, there are records for only half a dozen species and it has not been reported from Nicaragua so far (Flowers 1996). This would be the first country record of *Paria*, corresponding to one species that was known from Guatemala and Panama, thus providing an intermediate occurrence that suggests a more continuous distribution in tropical Mesoamerica. The specimens from Nicaragua conform to the type and description of *P. nigritarsis*, except for their pale tarsi. However, this seems to be a polymorphic trait in the species, considering that Jacoby (1891) already reported that the specimens he had seen from Panama also had pale tarsi. The cephalic traits of this species, including the narrow supraocular furrow and the broad, flat area of frontoclypeus between antennae clearly refer the species to the genus *Paria*. We provide with illustrations of habitus (Fig. 6b) and spermatheca (Fig. 5g).

***Rhabdopterus uncotibialis*** **(Blake, **1976a**)**

(Figs. 3d, 4d, 5h)

New species record: **JGZC:** (1) female (JGZC-1226), Atlántico Norte, cra. a Siuna, Empalme-Coperna, Puente Baka, June 2009, Fr. LePont leg., *Rhabdopterus uncotibialis* (Blake) J. Gómez-Zurita det.; (2) female (JGZC-1378), Jinotega, Volcán Yali (norte), 13.266881 -86.1724001, 4 September 2010, J.-M. Maes leg., *Rhabdopterus uncotibialis* (Blake) J. Gómez-Zurita det.; (3) male (JGZC-1401), Matagalpa, Selva Negra, 12°59′53.13″N 85°53′38.78″W, November 2010, J.-M. Maes leg., *Rhabdopterus uncotibialis* (Blake) J. Gómez-Zurita det. **MEL:** (1) 1 ex., #263, Matagalpa, Selva Negra, 12.996111 -85.908330, 1300 m, 5–7 March 2001, J.-M. Maes, J. Peña & B. Téllez leg.; (2) 1 ex., Región Autónoma de la Costa Caribe Norte, 8 km E Bonanza, Centro de Información y Comunicación Regional para el Trópico Húmedo (CICABO), 14.013335 -84.525334, 100 m, 5 April 2000, on *Theobroma cacao*, J.-M. Maes & B. Hernández leg.; (3) 1 ex., *idem*, 15 June 2000, on *Persea americana*, Y. Dixon leg.; (4) 1 ex., *idem*, 28 June 2000, on *Theobroma cacao*; (5) 3 exx., *idem*, 4 July 2000; (6) 2 exx., *idem*, 14 July 2000; (7) 6 exx., *idem*, 19–20 July 2000; (8) 1 ex., *idem*, 21 July 2000, on *Canavalia ensiformis*; (9) 5 exx., #11 and #259, *idem*, 2 August 2000, on *Theobroma cacao*, *Rhabdopterus sp.* R. Westerduijn det. 2020; (10) 1 ex., *idem*, 21 August 2000; (11) 1 ex., *idem*, 23 October 2000; (12) 1 ex., *idem*, 8 November 2000, sobre canela; (13) 1 ex., *idem*, 20 November 2000, on *Theobroma cacao*; (14) 1 ex., *idem*, 12 December 2000, sobre aguacate; (15) 1 ex., *idem*, 19 December 2000, sobre guanabana.

*Rhabdopterus* Lefèvre, 1885 is a diverse genus in both the Nearctic and Neotropical regions, of relatively imprecise boundaries and with up to thirteen species distributed in Central America, six of them possibly in Nicaragua (Maes and Staines 1991; Flowers 1996). Most species were described by Jacoby (1882) as *Rhabdophorus*, and a few were described by Blake (1976a), who interpreted them as semicostate *Colaspis* (Flowers 1996). The species of *Rhabdopterus* often present species-specific color patterns in their antennae, with different sets of alternating pale and dark antennomeres. The specimens from Nicaragua clearly have the seventh (and to some extent the sixth as well) and three apical antennomeres dark, which is a very unusual combination. As far as we know, this pattern has only been described for *R. uncotibialis*, a species currently known from Panama thus far, and the Nicaraguan specimens (Fig. 4d) fit the description of this species in every other detail, including the size above 5.5 mm. Interestingly, it is not possible to reach this species using Blake’s (1976a) key because she considered different length proportions of pronotum and elytra as observed in the Nicaraguan specimens, which have elytra three or more times longer than pronotum. This trait would lead to *R. panamensis* (Blake, 1976a) instead, which is admittedly similar to *R. uncotibialis*, but clearly smaller (< 5 mm) and lacks the characteristic color pattern of the antennae (Blake 1976a). Moreover, the penis of the Nicaraguan male specimen (Fig. 3d) has the characteristic angulate preapical margin of *R. uncotibialis*, instead of the gradually narrowed apical margin that Blake (1976a) described for *R. panamensis*. The availability of females allows illustrating the spermatheca of the species (Fig. 5h), and our new records considerably expand the range of the species northward.

***Spintherophyta ignita*** **(Lefèvre,** 1877 **)**

(Figs. 1e, 5i)

New species record: **JGZC:** female (JGZC-1304), Río San Juan, El Castillo, refugio Bartola, 10°58′37.19″N 84°20′12.33″W, 79 m, 3–6 November 2008, J.-M. Maes leg., *Spintherophyta ignita* (Lef.) J. Gómez-Zurita det. 2022.

Lefèvre (1877) described *Chrysodina ignita*, one of the largest (4.0–4.5 mm) species of *Chrysodina*, of golden purple color, with metallic legs and four basal antennomeres pale, darkened dorsally on scape. Jacoby (1881) commented on the possibility that this species could be the same as *C. fuscitarsis* Lefèvre, 1877, also metallic purple dorsally, but slightly smaller (3.5–4.0 mm) and with five basal antennomeres fulvous and tarsi and apex of tibiae darker. Bechyné (1951) recognized dark violaceous forms of *C. ignita* in Costa Rica (= ab. supraviolacea). The specimen from Nicaragua, which fits the description of *Spintherophyta*, is a large (4.7 mm) female, dark violaceous blue, with four pale antennomeres basally and scape with a dark dorsal spot, and can be tentatively classified as *Spintherophyta ignita* (Lefèvre). We document it here (Fig. 1e) as well as the type of spermatheca (Fig. 5i) to assist its interpretation in the future. This species has been reported from Mexico and Costa Rica (Bechyné 1951; Flowers 1996) and the Nicaraguan record would help bridging the gap between these distant localities.

***Spintherophyta minuta*** **(Jacoby, **1881**)**

(Figs. 1f, 3e, 5j)

New species record: **JGZC:** (1) female (JGZC-2612), Rivas, Cárdenas, Finca Isla Vista, 11.23345 -85.55123, 56 m, 6 February 2012, A. Cardoso, G. De la Cadena, J.-M. Maes & A. Papadopoulou leg., *Spintherophyta minuta* (Jac.) J. Gómez-Zurita det. 2022; (2) female (JGZC-2626), Rivas, Cárdenas, Finca Sierra Serena, 11.23527 -85.55388, 69 m, 7 February 2012, A. Cardoso, G. De la Cadena, J.-M. Maes & A. Papadopoulou leg., *Spintherophyta minuta* (Jac.) J. Gómez-Zurita det. 2022; (3) female (JGZC-1308) and males (JGZC-1298, JGZC-1310), Rivas, Sapoa, Finca Guadalupe, 11.179983 -85.6807925, 290 m, 15 September 2009, J.-M. Maes leg., *Spintherophyta minuta* (Jac.) J. Gómez-Zurita det. 2022; (4) female (JGZC-2631), Rivas, Sapoa, Finca Guadalupe, 11.18847 -85.67619, 157 m, 9 February 2012, A. Cardoso, G. De la Cadena, J.-M. Maes & A. Papadopoulou leg., *Spintherophyta minuta* (Jac.) J. Gómez-Zurita det. 2022.

Jacoby (1881, p. 110) described *Chrysodina minuta* from Guatemala, not to be confounded with *Lamprosphaerus minutus* described from the same country (p. 113), the latter having been already reported from Nicaragua (Maes and Staines 1991). Relative to the former, the author expressed doubts about its correct generic placement, mainly because of its relatively elongate shape and long prosternum, which did not fit his concept of *Chrysodina* (Jacoby 1890). Lefèvre (1889a) transferred Jacoby’s taxon to *Lamprosphaerus* without justification, but Jacoby (1890) was not satisfied with this decision, highlighting that the enlarged apical antennomeres of his species were not characteristic of *Lamprosphaerus*. Bechyné (1953) proposed a new combination with *Nodonota*, and years later to *Antitypona*, but without any justification (Bechyné 1955). Flowers (1996) treated most *Chrysodina* described by Jacoby as *Spintherophyta*, including *C. minuta*. The specimens collected in Nicaragua (Fig. 1f) agree well with the type of *C. minuta*, also in the shape of their antennae, with short antennomeres 3 and 4 and a markedly enlarged second antennomere, characteristic of Central American *Spintherophyta*; they also show clear microreticulation on head and pronotum, even though the dorsal tinge is of a stronger green and blue hue compared with the type. Thus, we tend to agree with Flowers’ (1996) assessment for this species and retain it in *Spintherophyta*, and take the opportunity to illustrate the penis (Fig. 3e) and spermatheca (Fig. 5j) of this species for the first time. *S. minuta* is only known from Guatemala, and the Nicaraguan records, all found in the south of the country, represent a remarkable expansion of the known range of the species.

***Talurus tortonesei*** **(Bechyné, **1957**)**

(Figs. 3f, 4e, 5k)

Confirmation records: **JGZC:** (1) 3 females (JGZC-1205, JGZC-1207, JGZC-1217) and 2 males (JGZC-1208, JGZC-1209, JGZC-1219), Granada, Reserva Silvestre Privada Domitila, May 2010, J.-M. Maes leg., *Talurus tortonesei* Bech. J. Gómez-Zurita det. 2022; (2) 7 females (JGZC-1943, JGZC-1945 to JGZC-1950), Granada, Reserva Natural Lagunas de Mecatepe y Río Manares, Hacienda Las Plazuelas, 11°46′16.59″N 85°57′41.68″W, 62 m, June 2011, J.-M. Maes leg., *Talurus tortonesei* Bech. J. Gómez-Zurita det. 2022. **MEL:** (1) 1 ex., Chinandega, Pikin Guerrero, 12.666235 -86.973671, 560 m, June 1994, on *Phaseolus reticulatus*, J. Munguia leg.; (2) 3 exx., Granada, Volcán Mombacho, Finca San Joaquín, 11.825672 -85.988872, 650 m, trampa Malaise en cultivo orgánico de café, 30 June 1998, J.-M. Maes leg.; (3) 1 ex., Granada, Volcán Mombacho, 11.827567 -85.963137, 1100 m, July 2002, bosque de neblina, L. Huez leg.; (4) 8 exx., Granada, Reserva Domitila, 11.701478 -85.949158, 55 m, May 2005, J.-M. Maes leg.; (5) 5 exx., #5, *idem*, 10–14 June 2007, *Talurus tortonesi* R. Westerduijn det. 2020 and W. Flowers det. 2020; (6) 3 exx., #151, *idem*, June 2008, *Talurus tortonesi* W. Flowers det. 2020; (7) 1 ex., León, cra. a Poneloya, El Carmen, 12.350003 -86.966653, 10 m, 1 July 1983, on *Pithecelobium dulce* (Mimosaceae), J.-M. Maes leg., Eumolpinae I. Askevold det. 1987; (8) 1 ex., #168, León, 12.437571 -86.874655, 110 m, 22 June 1986, sobre pipian, R. Cisneros leg., *Talurus salvini* I. Askevold det. 1987, *Talurus tortonesi* W. Flowers det. 2020; (9) 3 exx., León, 12.437571 -86.874655, 110 m, May 1988, J.-M. Maes leg.; (10) 6 exx., *idem*, July 1989; (11) 1 ex., *idem*, July 1992; (12) 2 exx., #166, *idem*, May 1993, J.-M. Maes & J. Téllez leg., *Talurus tortonesi* W. Flowers det. 2020; (13) 1 ex., *idem*, June 1993; (14) 2 exx., *idem*, August 1997, J.-M. Maes leg.; (15) 1 ex., León, Museo Entomológico, 12.437571 -86.874655, 110 m, June 1995, J.-M. Maes leg.; (16) 1 ex., *idem*, May 1999; (17) 1 ex., *idem*, 5 June 2000; (18) 1 ex., León, Centro de Iniciativa Medio Ambiental (CIMAC), 12.432740 -86.873283, 96 m, 20 June 2000, trampa de luz, M. Torres leg.; (19) 8 exx., *idem*, 24 June 2000, M. Torres & B. Hernández leg.; (20) 1 ex., León, Colegio Manuel Ignacio Lacayo, 12.435225 -86.902045, 83 m, 28 May 1988, on *Zea mays*, J.-M. Maes leg.; (21) 3 exx., León, La Paz Centro, 12.343411 -86.676218, 80 m, 19 May 1995, plaga de soja; (22) 1 ex., Managua, La Morita, 12.114993 -86.236174, 160 m, 29 June 1959, sobre hoja de frijol, J. Morales leg.; (23) 1 ex., Managua, Universidad Nacional Agraria, La Calera, 12.147945 -86.161338, 56 m, 23 July 1962, sobre frijol, L. Saenz leg.; (24) 1 ex., #145, Managua, Mateare, 12.234602 -86.431384, 70 m, 27 October 1994, on *Jatropha curcas*, C. Grimm leg., *Talurus tortonesi* W. Flowers det. 2020; (25) 278 exx, #1–#4, Managua, cra. Managua-León, km 23, 12.268424 -86.480057, 50 m, 27 June 1997, sobre *Gliricidia sepium*, J.-M. Maes & V. Thompson leg., *Talurus tortonesi* W. Flowers det. 2020; (26) 5 exx., Managua, Finca La Chinampa, 12.075308 -86.460169, 230 m, 28–29 September 2001, J.-M. Maes leg.; (27) 17 exx., Masaya, Las Flores, Finca Téllez, 12.003716 -86.020843, 100 m, June 1987, trampa de luz, J.-M. Maes leg. [one, #158, with: *Talurus tortonesi* W. Flowers det. 2020]; (28) 14 exx., #165, *idem*, June 1993, trampa de luz, Lecocq & Cantamessa leg., *Talurus tortonesi* W. Flowers det. 2020; (29) 4 exx., *idem*, sobre frijol; (30) 103 exx., #6–#7, #146 and #148–#150, *idem*, 30 May 1994, trampa de luz, J.-M. Maes & J. Hernández leg., *Talurus tortonesi* R. Westerduijn det. 2020, *Talurus tortonesi* W. Flowers det. 2020; (31) 1 ex., *idem*, 5 June 1994, J.-M. Maes, J. Téllez & J. Hernández leg., *Talurus tortonesi* R. W. Flowers det.; (32) 1 ex., *idem*, 15 June 1994, J.-M. Maes leg.; (33) 1 ex., *idem*, 8 July 1994, trampa de luz, J.-M. Maes & J. Hernández leg.; (34) 5 exx., *idem*, 18 June 1995, J. Hernández leg.; (35) 2 exx., *idem*, May 2000, M. Téllez leg.; (36) 3 exx., *idem*, June 2000, B. Téllez leg.; (37) 2 exx., #153, *idem*, 25 September 2003, S. Téllez leg., *Talurus tortonesi* W. Flowers det. 2020; (38) 1 ex., *idem*, June 2009, S. Téllez leg.; (39) 1 ex., Matagalpa, Matiguas, 12.838320 -85.460764, 300 m, 15 August 1983, en pasto, A. Castillo leg.; (40) 10 exx., #272 and #273, Nueva Segovia, Santa Clara, San Nicolás, 13.750511 -86.239781, 670 m, 24 Jun 2008, trampa de luz, F. Muñoz leg.; (41) 2 exx., #167, Rivas, Escuela Internacional de Agricultura y Ganadería, 11.438137 -85.834226, 67 m, 13 August 1971, sobre maíz, L. Saenz leg., *Talurus tortonesi* W. Flowers det. 2020.

This species of *Talurus* Lefèvre, 1889b, which was originally described from Corinto, a coastal locality of Chinandega (Nicaragua), is common and widespread in Nicaragua, although Maes and Staines (1991) missed reporting it in their catalog. While this is not a proper new country record, we take the opportunity to amend the previous catalog with our own records of this species in Nicaragua, including illustrations of habitus (Fig. 4e) and genitalia (Figs. 3f and 5k). This species is also important for another reason. *T. rugosus* (Jacoby, 1882), described from Mexico, has been reported as a pest of different crops across Central America (e.g., Coto et al. 1995), and there has been a trend to automatically refer all *Talurus* found attacking crops to this species, including in Nicaragua (e.g., Maes and Staines 1991). However, based on our assessment, all the available specimens of *Talurus* from Nicaragua, which display quite a range of chromatic alternatives, actually belong to *T. tortonesei* Bechyné, and perhaps other country records also need a reassessment (e.g., Van Roie et al. 2019). Bechyné (1957) detailed the main differences between the two species, where the most remarkable would include the preapical enlargement of protibiae in *T. tortonesei*, against the straight tibiae of *T. rugosus*, but also the finer, denser punctation on pronotum and a less transverse pronotum in the case of *T. tortonesei*.

***Tijucana vitticollis*** **(Jacoby, 1882)**

(Figs. 2c, 6c)

New genus and species record: **JGZC:** female (JGZC-1305), Río San Juan, El Castillo, Refugio Bartola, 10°58′37.19″N 84°20′12.33″W, 79 m, 3–6 November 2009, J.-M. Maes leg., *Tijucana vitticollis* (Jac.) J. Gómez-Zurita det. 2022. **MEL:** 1 ex., #223, Río San Juan, Refugio Bartola, 10.972221 -84.33889, 40 m, 16–20 June 2007, J.-M. Maes leg.

*Tijucana* Bechyné, 1957 is a small genus that was proposed based on eyes strongly emarginate internally and reduced anterior prosternal lobes, and it only contains three known species (Bechyné 1958): one from Brasil (State of Rio de Janeiro), one from Bolivia, and one that was known from Panama and later found in Costa Rica (Flowers 1996). The presence of *T. vitticollis* in rainforests of the south of Nicaragua (Fig. 6c), close to the border with Costa Rica, slightly expands the range of the genus further north. Interestingly, one of the specimens, a female, has abnormally formed apex of right mesotibia and corresponding mesotarsus, where a notable case of trifid tarsal schistomelia can be observed (Fig. 2c). Schistomelia is the division of an appendix in two or more growth axes, and while this type of abnormality typically affects the antennae of many insects, there are relatively few reports of this process affecting the legs of Coleoptera (Ortuño and Ramos Abuín 2008), which makes this observation particularly interesting.

***Typophorus apicicornis*** **(Jacoby,** 1891 **)**

(Figs. 5l, 6d)

New species record: **JGZC:** female (JGZC-2591), Managua, Montelimar, Reserva Natura, 11.87659 -86.88130, 36 m, 24 January 2012, J. Gómez-Zurita, A. Cardoso, G. De la Cadena & A. Papadopoulou leg., *Typophorus apicicornis* Jac. J. Gómez-Zurita det. 2022.

This species of *Typophorus* Erichson, 1847, was previously known only from Panama (Bechyné 1953; Flowers 1996), and it is relatively easy to identify based on chromatic pattern. The individuals are dark reddish brown with four darker spots on elytra, which are faint but visible in the Nicaraguan specimen, and the antennae have antennomeres 6–10 black and the remaining antennomeres pale, including the apical one (Fig. 6d). The legs of *T. apicicornis* are dark reddish brown with apex of tibiae and femora black, a contrast that also occurs in the Nicaraguan specimen although it is difficult to appreciate because of its generalized dark color. Punctation of the Nicaraguan specimen also fits the type. The only specimen available for study is a female and the spermatheca is figured for future reference (Fig. 5l). As with most species reported in this work, the presence of the species in Nicaragua greatly expands the range of the species in a significant part of Central America.

***Typophorus cyanipennis*** **(Lefèvre, **1876**)**

(Figs. 5m, 6e)

New species record: **JGZC:** female (JGZC-1387), Managua, Montelimar, Reserva Natura, 11.862763 -86.5117021, 69 m, 9 October 2010, J.-M. Maes leg., *Typophorus cyanipennis* Lef. J. Gómez-Zurita det. 2022. **MEL:** 1 ex., #247, Managua, El Crucero, 11.980569 -86.311052, 900 m, 27 June 1997, J.-M. Maes & V. Thompson leg., *Typophorus cf. mexicanus* R. Westerduijn det. 2020.

This originally Mexican species of *Typophorus* has been recognized as allied to another Mexican and Guatemalan species, *T. mexicanus* Jacoby, 1876 (Jacoby 1882). While it was initially proposed that the reddish legs of the former (at least on femora basally) could distinguish them compared with the dark metallic legs of the latter (Jacoby 1876, 1882), it was subsequently recognized that *T. mexicanus* could show variability in this trait as well (Jacoby 1891), thus complicating the separation of both species. Both *T. cyanipennis* and *T. mexicanus* share their very characteristic chromatic pattern and a tenuous, nearly imperceptible punctation on head and thorax, and faint, sparse punctation on elytra, and they may be indeed conspecific. Jacoby (1891) struggled to keep them separated on the basis of supposedly longer antennae in *T. mexicanus*. While this issue is settled, the specimens from Nicaragua, including a 4.0 mm long female (Fig. 6e) conforms better to the original description and chromatic traits of *T. cyannipennis*, also in the smaller size (Lefèvre gave 4.5–6.0 mm for his taxon, and Jacoby 5.3 mm), and considering that Jacoby’s description was published nearly at the end of 1876 (5th December), it is possible that Lefèvre’s taxon would have precedence in case of an eventual synonymy were decided. The spermatheca of one Nicaraguan specimen is figured (Fig. 5m).

***Typophorus subbrunneus*** **(Jacoby, 1882)**

(Figs. 3g, 5n, 6f)

New species record: **JGZC:** (1) female (JGZC-1896), León, Jardín Botánico (bosque de galería), 12.433378 -86.9140889, 50 m [919], 4 August 2011, J.-M. Maes leg., *Typophorus subbrunneus* Jac. J. Gómez-Zurita det. 2022; (2) female (JGZC-1389), Managua, Montelimar, Reserva Natura, 11.866200 -86.5064809, 74 m, 8 October 2010, J.-M. Maes leg., *Typophorus subbrunneus* Jac. J. Gómez-Zurita det. 2022; (3) female (JGZC-2611) and male (JGZC-2614), Rivas, Cárdenas, Finca Isla Vista, 11.23345 -85.55123, 56 m, 6 February 2012, A. Cardoso, G. De la Cadena, A. Papadopoulou & J.-M. Maes leg., *Typophorus subbrunneus* Jac. J. Gómez-Zurita det. 2022; (4) female (JGZC-2600), *idem*, 8 February 2012, A. Cardoso, G. De la Cadena, A. Papadopoulou & J.-M. Maes leg., *Typophorus subbrunneus* Jac. J. Gómez-Zurita det. 2022; (5) male (JGZC-2625), Rivas, Cárdenas, Finca Sierra Serena, 11.23527 -85.55388, 69 m, 7 February 2012, A. Cardoso, G. De la Cadena, A. Papadopoulou & J.-M. Maes leg., *Typophorus subbrunneus* Jac. J. Gómez-Zurita det. 2022; (6) male (JGZC-2630), Rivas, Sapoa, Finca Guadalupe, 11.18847 -85.67619, 157 m, 9 February 2012, A. Cardoso, G. De la Cadena, A. Papadopoulou & J.-M. Maes leg., *Typophorus subbrunneus* Jac. J. Gómez-Zurita det. 2022.

This tiny (2.1–2.6 mm) species of *Typophorus* is relatively easy to recognize because of its brownish color, when many species of *Typophorus* have bright tones, the impunctate pronotum, and the apex of tibiae and tarsi paler than the remainder of legs (Fig. 6f). The species was reported from Belize and Guatemala (Jacoby 1882; Clavareau 1914; Bechyné 1953; Flowers 1996), and its presence in Nicaragua expands considerably its range south. Together with *T. apicicornis* reported above, this species presents some structural facial differences relative to typical *Typophorus*. Most notably, they both have a wide, subparallel, uninterrupted, and, at most, slightly depressed transition between frons and clypeus, lacking the characteristic transverse furrow dividing the frontoclypeus in *Typophorus* and more in line with the typical facial structure of *Paria* (Jacoby 1882). However, these two species have wide, posteriorly enlarged supraocular sulci, characteristic of *Typophorus* (and lacking in *Paria*, for example), and for the time being, even though we acknowledge that they will likely require a new generic combination, we chose to treat them in their original genus for stability. We illustrate male genitalia (Fig. 3g) and spermatheca (Fig. 5n) of this species for the first time.
